# Stratified randomization controls better for batch effects in 450K methylation analysis: a cautionary tale

**DOI:** 10.3389/fgene.2014.00354

**Published:** 2014-10-13

**Authors:** Olive D. Buhule, Ryan L. Minster, Nicola L. Hawley, Mario Medvedovic, Guangyun Sun, Satupaitea Viali, Ranjan Deka, Stephen T. McGarvey, Daniel E. Weeks

**Affiliations:** ^1^Department of Biostatistics, Graduate School of Public Health, University of PittsburghPittsburgh, PA, USA; ^2^Department of Human Genetics, Graduate School of Public Health, University of PittsburghPittsburgh, PA, USA; ^3^Department of Chronic Disease Epidemiology, School of Public Health, Yale UniversityNew Haven, CT, USA; ^4^Department of Environmental Health, University of Cincinnati College of MedicineCincinnati, OH, USA; ^5^Faculty of Medicine, National University of SamoaApia, Samoa; ^6^Department of Epidemiology, International Health Institute, Brown University School of Public HealthProvidence, RI, USA

**Keywords:** array data, batch effects, DNA methylation, epigenetics, obesity, study design

## Abstract

**Background:** Batch effects in DNA methylation microarray experiments can lead to spurious results if not properly handled during the plating of samples.

**Methods:** Two pilot studies examining the association of DNA methylation patterns across the genome with obesity in Samoan men were investigated for chip- and row-specific batch effects. For each study, the DNA of 46 obese men and 46 lean men were assayed using Illumina's Infinium HumanMethylation450 BeadChip. In the first study (Sample One), samples from obese and lean subjects were examined on separate chips. In the second study (Sample Two), the samples were balanced on the chips by lean/obese status, age group, and census region. We used methylumi, watermelon, and limma R packages, as well as ComBat, to analyze the data. Principal component analysis and linear regression were, respectively, employed to identify the top principal components and to test for their association with the batches and lean/obese status. To identify differentially methylated positions (DMPs) between obese and lean males at each locus, we used a moderated *t*-test.

**Results:** Chip effects were effectively removed from Sample Two but not Sample One. In addition, dramatic differences were observed between the two sets of DMP results. After “removing” batch effects with ComBat, Sample One had 94,191 probes differentially methylated at a *q*-value threshold of 0.05 while Sample Two had zero differentially methylated probes. The disparate results from Sample One and Sample Two likely arise due to the confounding of lean/obese status with chip and row batch effects.

**Conclusion:** Even the best possible statistical adjustments for batch effects may not completely remove them. Proper study design is vital for guarding against spurious findings due to such effects.

## Introduction

DNA methylation is a vital type of epigenetic modification which usually occurs in CpG-rich regions in mammals and is involved in regulating gene expression and silencing (Selaru et al., 2009; Sharma et al., 2010). Altered methylation levels, such as those due to environmental factors and lifestyle, may play a role in a variety of disease processes. For instance, many studies have revealed association of aberrant DNA methylation with diseases such as cancers (Karpiński et al., 2008; Feinberg and Irizarry, 2010; Hansen et al., 2011), obesity (Feinberg et al., 2010; Wang et al., 2010; Xu et al., 2013; Dick et al., 2014), and rheumatoid arthritis (Liu et al., 2013). High-throughput technologies, such as microarray and sequencing-based DNA methylation profiling, have been developed to facilitate the investigation of gene expression, gene regulation, and epigenetic interactions between cells and environment.

The Infinium HumanMethylation450 BeadChip (Illumina, San Diego, CA) is one of the most commonly used epigenome-wide methylation profiling platforms. It covers 99% of RefSeq genes and 96% of CpG islands, with additional coverage in island shores and the regions flanking them. This technology interrogates more than 485,000 methylation sites per sample, and each chip can accommodate 12 samples in a 2 column by 6 row matrix. Thus, samples in large studies are often assayed across many different individual chips processed at different times, which may result in batch effects (Johnson et al., 2007; Leek et al., 2010; Sun et al., 2011; Yan et al., 2012; Harper et al., 2013). Batch effects are non-biological variations that are related to experimental factors, such as laboratory conditions, experiment time, reagent lots, laboratory personnel differences, and chip position. Batch effects are a major problem when they are correlated with the outcome or predictors of interest. Without appropriate correction measures, batch effects may lead to inaccurate conclusions (false positives) or increased variability and significant reduction in power to detect true biological signals (Baggerly et al., 2004; Akey et al., 2007; Leek and Storey, 2007). Moreover, undetected batch effects can lead to substantial misallocation of resources and lack of reproducibility (Baggerly et al., 2008). Batch effects that affect different probes in different ways cannot be removed by normalization methods that adjust for global properties of measurements. Special techniques like ComBat (Johnson et al., 2007) are employed to adjust for batch effects; however, their effectiveness depends on the study design. Although batch effects cannot be fully eliminated from even a perfectly designed study, Hu et al. (2005) stressed that the key step to addressing batch effects and other technical artifacts in high-throughput data is careful study design. In a case–control study, the cases and controls should be equally distributed across the factors considered to be a batch effect (Hu et al., 2005). For example, Liu et al. (2013) found an unanticipated association between their methylation data and assay date, which was the result of an unbalanced distribution of cases and controls across those dates. Similarly, Harper et al. (2013) found that when samples are not randomized across chips, then even powerful techniques like ComBat could not fully remove the batch effects, hence leading to an excess of apparently differentially methylated probes. However, randomization does not ensure equal allocation of cases and controls across the chips, especially with small samples.

In this study, we present the findings of two pilot studies examining DNA methylation profiles in Samoan obese and lean young male adults to illustrate how chip-specific effects can lead to spurious results when an unbalanced study design is employed during the plating of samples.

### Why two pilot studies?

The original objective of the first pilot study (Sample One) was to examine DNA methylation patterns across the genome in 46 obese and 46 lean male Samoans to identify epigenetic loci associated with obesity. We carried out standard quality control steps. When we examined box plots of raw and normalized β-values for each participant ordered by chip position, we observed patterns that suggested chip and row effects, and we tried to remove those batch effects using ComBat. To determine whether the adjustment worked, we examined plots of the technical replicates, and the agreements in values between the replicates seemed to show that ComBat had indeed removed the batch effects. Therefore, we moved ahead and tested for probes that were differentially methylated between the lean and obese individuals. The results seemed promising, and hence an abstract was written and accepted for presentation at the 2013 meeting of the American Society of Human Genetics (Buhule et al., 2013).

**Figure 1:**
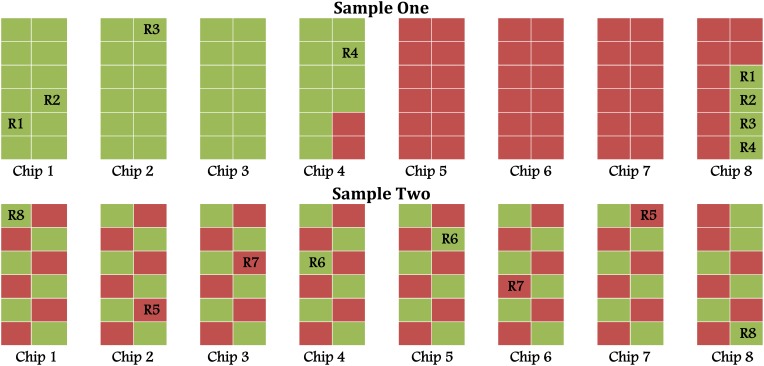
**Chip layout in Sample One (unbalanced) and Sample Two (balanced); green and red represent lean and obese, respectively**.

However, we were concerned about the apparent excess of significant probes and uncomfortable with how the samples had been arrayed on the chips in Sample One (upper panel of Figure 1). To assess whether the layout of samples might have had an effect on our results—one that could not be removed by ComBat—and to attempt to replicate the differentially methylation probes from Sample One, we performed a second pilot study (Sample Two). As in Sample One, 46 lean and 46 obese Samoan men were examined. The samples were carefully balanced across the chips to avoid confounding the outcome and predictor variables with the layout (lower panel of Figure 1). We followed the same analysis steps as in the first pilot study.

## Materials and methods

### Sample collection and DNA extraction

Two sets of 92 young (25–40 years) males each were drawn, using stratified sampling by census region, from the same Samoan population. This represents a subsample from our larger genome-wide association study of adiposity-related traits, the design of which is described in Hawley et al. (2014). The informed consent and research protocols were approved by both the Brown University Institutional Review Board (protocol #0903992671) and the Health Research Committee of the Samoan Ministry of Health. The demographic characteristics of the two sets are summarized in Table 1. For each set, DNA was extracted from the peripheral whole blood of 46 obese (body mass index ≥ 32 kg/m^2^ and abdominal circumference ≥ 92.5 cm) and 46 lean (body mass index <26 kg/m^2^ and abdominal circumference <92.5 cm) Samoan males. The threshold of 92.5 cm was chosen because it was the overall sample median for waist circumference among this age group. As Table 1 shows, while both Samples have mean ages in the 30's, Sample Two has a lower mean age than Sample One, both in the lean group (*t*-test *p*-value 0.003; Cohen's *d* effect size 0.64) and in the obese group (*p*-value 0.005; effect size 0.60); comparing the obese men, Sample Two also has a lower mean BMI (*p*-value 0.033; effect size 0.46) and abdominal circumference (*p*-value 0.005; effect size 0.61) than Sample One (Cohen, 1992).

**Table 1:** **Study sample demographics**.

**Variable**	**Sample One**		**Sample Two**	
	**Lean *n* = 46**	**Obese *n* = 46**	**Lean *n* = 46**	**Obese *n* = 46**
Age (years), x (*s*)	33.4 (4.1)	35.0 (3.6)	30.5 (4.9)	32.5 (4.5)
BMI (kg/m^2^), x (*s*)	24.2 (1.4)	37.0 (5.2)	24.3 (1.3)	35.1 (2.6)
Abd. Circumf. (cm), x (*s*)	81.9 (4.5)	113.2 (12.7)	81.0 (4.0)	107.1 (6.4)
**CENSUS REGIONS**				
Apia, *n* (%)	9 (19.5)	9 (19.6)	9 (19.6)	13 (28.3)
NW Upolu, *n* (%)	15 (32.6)	15 (32.6)	15 (32.6)	16 (34.8)
Rest of Upolu, *n* (%)	12 (26.1)	12 (26.1)	12 (26.1)	14 (30.4)
Savai‘i, *n* (%)	10 (21.7)	10 (21.7)	10 (21.7)	3 (6.5)

Blood was drawn while fasting the morning after anthropometric measures were taken. Methylation levels were assayed using the Infinium HumanMethylation450 BeadChip array (Illumina, San Diego, CA) at the Genomics, Epigenomics and Sequencing Core at the University of Cincinnati. For each sample, a total of 0.5–1 μg intact genomic DNA as measured by Qubit fluorometer (Lifetech, Grand Island, NY) was bisulfite modified by using Zymo EZ DNA methylation kit (Irvine, CA). The Illumina recommended incubation protocol (16 cycles of 95°C for 30 s, 50°C for 60 min) was used for the DNA bisulfite conversion. Methylated cytosine in a CpG site resists bisuflite modification and remains cytosine. In contrast, unmethylated cytosine is modified to uracil and converted to thymine in the subsequent amplification step.

Using Infinium HumanMethylation450 BeadChip kit, the bisulfite converted DNA is then denatured, isothermally amplified, enzymatically fragmented, and purified by precipitation. The resuspended DNA fragments were hybridized onto the chip with the beads attached to specific probes. After washing, a single-base extension to differentiate methylated cytosine (still cytosine) and unmethylated cytosine (converted to thymine) followed by staining of the BeadChip were performed. The cy5/cy3-stained BeadChip was then coating-protected and scanned on Illumina iSCAN to generate methylation raw data.

Figure 1 displays the layout of samples on different chips for the two data sets as well as the locations of eight samples technically replicated in pairs (R1–R8). In the first pilot study (Sample One), samples were “lumped together”—that is, the obese were plated first, then the lean, and lastly the technical replicates (Upper panel of Figure 1). For the second pilot study (Sample Two), the samples were balanced by lean/obese status across chips and rows (Lower panel of Figure 1). Additionally samples were arranged so that each chip carried the same proportion of participants from each Samoa census region and an equal number above and below the median age. This design should minimize the probability of confounding between biological and batch effects.

### Statistical analyses

**Figure 2:**
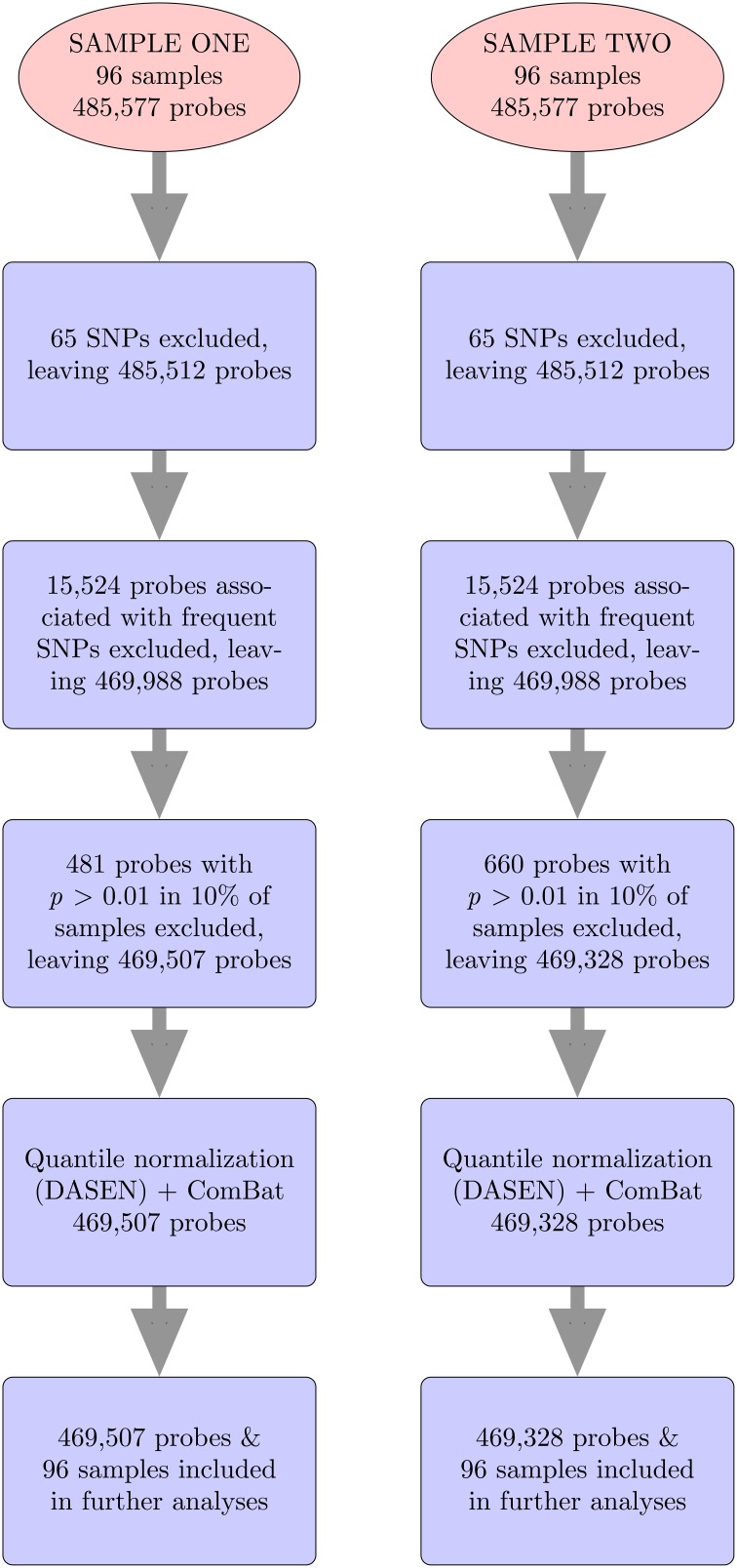
**450K methylation array data analysis pipeline**.

For both data sets, we followed same steps (Figure 2), using methylumi (Davis et al., 2013) and wateRmelon (Pidsley et al., 2013) R packages to analyze the data from the 450K Human Methylation Arrays. The raw ^*^.idat files were imported into R using the methylumIDAT() function from the methylumi package. For each data set, we excluded 65 SNP-containing probes that do not interrogate methylation and 15,524 probes associated with frequent SNPs using the East Asian (ASN) list from Touleimat and Tost's pipeline (Touleimat and Tost, 2012). We used East Asians because they represent the available population most similar to Samoans. We then performed filtering using the wateRmelon package to exclude any probes which have detection *p*-values > 0.01 in more than 10% of the samples or bead counts < 3 in more than 5% of samples. Samples with detection *p*-values > 0.01 in more than 10% of the probes would also have been excluded, but none met this threshold. Next, quantile normalization, which adjusts for background differences between Type I and Type II probes and does between-array normalization to these probes separately with no dye bias correction (DASEN), was performed. DASEN improves the ability to detect differentially methylated sites because Type I and Type II probes are known to perform differently (Pidsley et al., 2013). The *M*-values $\left(\log_{2}\left(\frac{\beta }{1-\beta }\right)\right)$ were then computed, where β-value is the ratio of the methylated probe intensity and the total signal intensity.

To identify any technical differences that may be emanating from a subset of samples or from sources not accounted for by quantile normalization—that is, batch effects—we examined box plots of the raw and normalized methylation values ordered by chip position to identify patterns in the data. We then adjusted the data for the chip and row batch effects using ComBat (Johnson et al., 2007). This method uses a parametric empirical Bayes framework to adjust data for batch effects and is robust to outliers in small sample sizes. ComBat was applied to *M*-values as these are preferred to β-values because β-values have been shown to have a non-constant variance (Du et al., 2010).

To determine whether ComBat reduced technical variation and effectively removed batch effects, first we examined plots of technical replicates. In particular, we calculated the average absolute differences in raw, pre- and post-ComBat normalized *M*-values, examined box plots of the raw, pre- and post-ComBat normalized *M*-values, and performed multidimensional scaling (MDS) and hierarchical clustering of the pre- and post-ComBat *M*-values. Secondly, principal component analysis (PCA) was performed on all samples to determine the top four principal components (PCs) present in the pre- and post-ComBat *M*-values. We tested for association between each PC and chip, row, or obese/lean group using linear regression.

To identify differentially methylated probes, we carried out analyses using the limma package (Smyth, 2005). Limma uses an empirical Bayes method to moderate the standard errors of the estimated log-fold changes. This leads to more stable inference and improved power because there is borrowing of strength from the body of probes when making inference about each individual probe (Smyth, 2004). Hence, the statistic used is called the moderated *t*-statistic, which was computed for each probe and then adjusted for multiple testing using the Benjamini-Hochberg method (Benjamini and Hochberg, 1997). The analysis was performed on both pre- and post-ComBat *M*-values.

## Results

### Exploratory analyses

**Figure 3:**
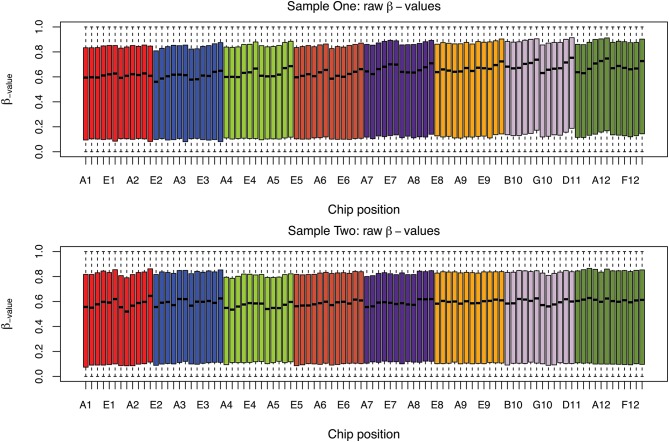
**Box plots of raw β-values by chip position; each color represents a chip**.

**Figure 4:**
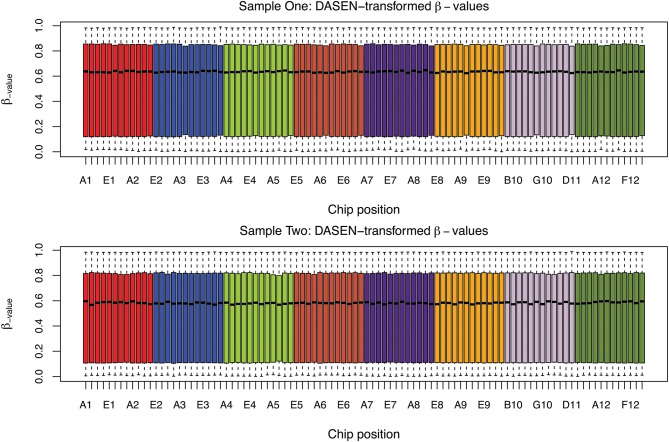
**Box plots of DASEN transformed β-values by chip position; each color represents a chip**.

The box plots of the β-values of raw and DASEN-transformed data from the two pipelines are presented in Figures 3, 4, respectively. The results in Figure 3 indicate a time-series–like trend of methylation levels (β-values) across and within the chips. In particular, the methylation levels increased from first row to sixth row within each chip. Normalization seem to have made this pattern less noticeable (Figure 4). Even though the batch effects are not as visually apparent, they still could be present, and indeed are as we will see below.

**Figure 5:**
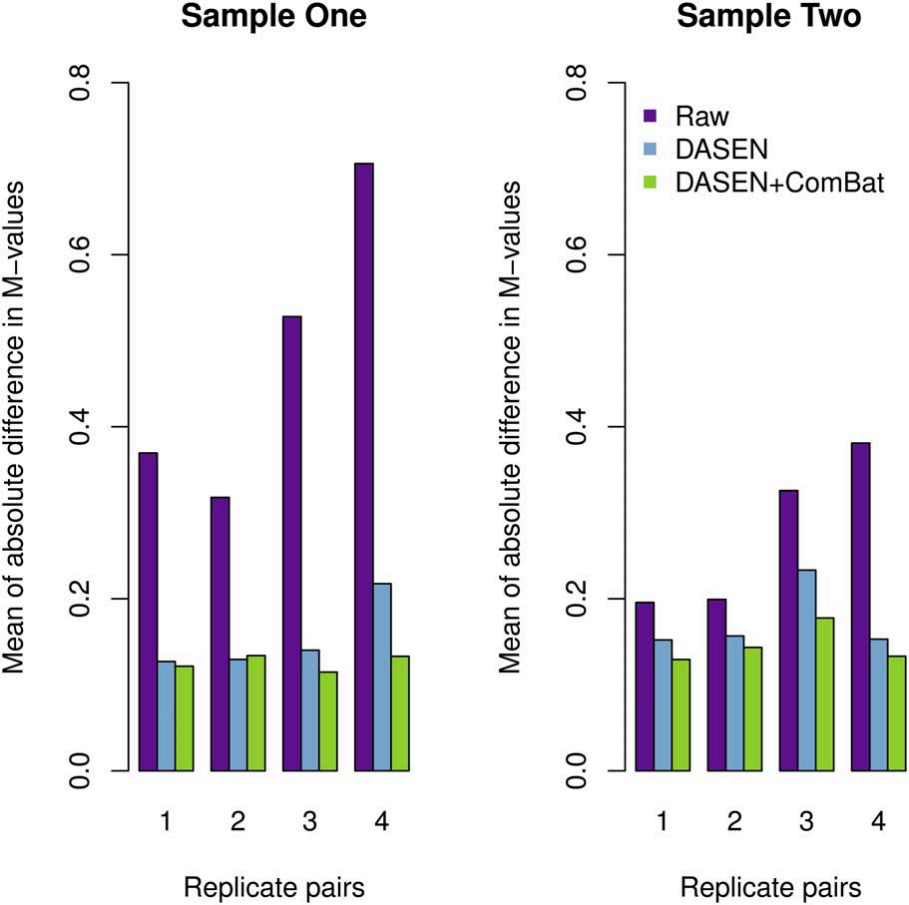
**Mean of absolute difference in *M*-values between four replicate pairs: the left and right panels represent Samples One and Two, respectively**.

**Figure 6:**
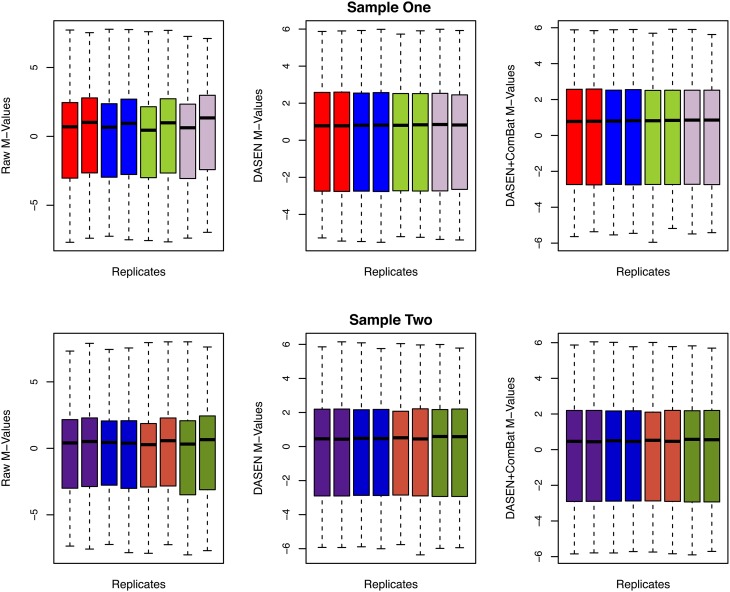
**Box plots of raw and transformed *M*-values for the four replicate pairs: upper and lower panels represent Samples One and Two, respectively, and each color represents a replicate pair**.

**Figure 7:**
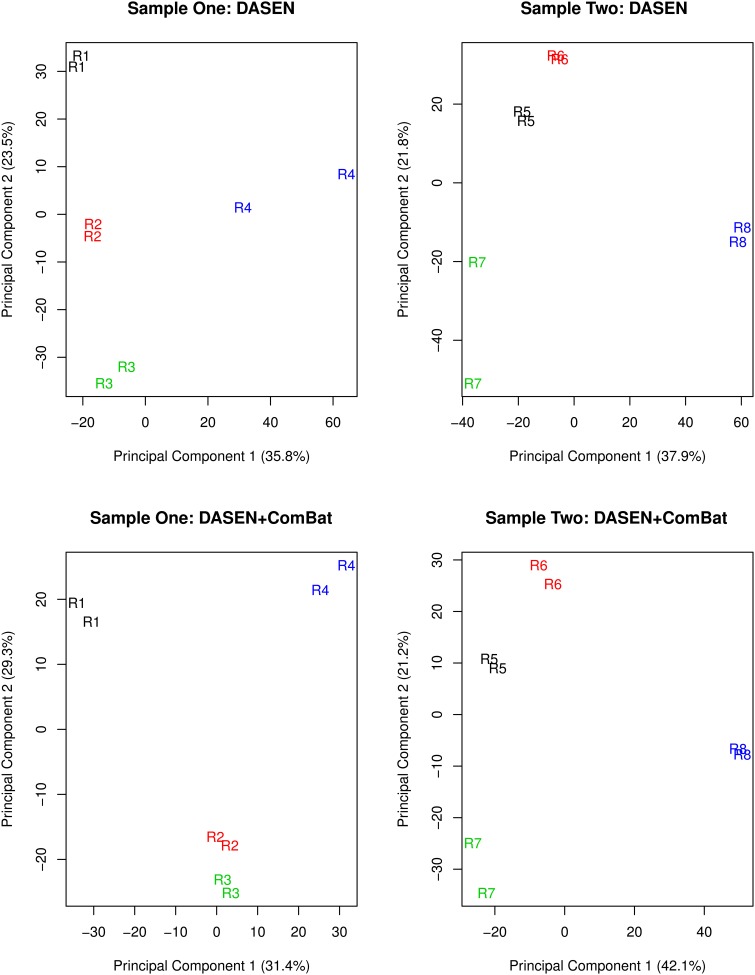
**Multidimensional scaling plots of technical replicates pre-ComBat (top row) and post-ComBat (bottom row)**. Duplicates are most closely correctly paired post-ComBat in both samples.

**Figure 8:**
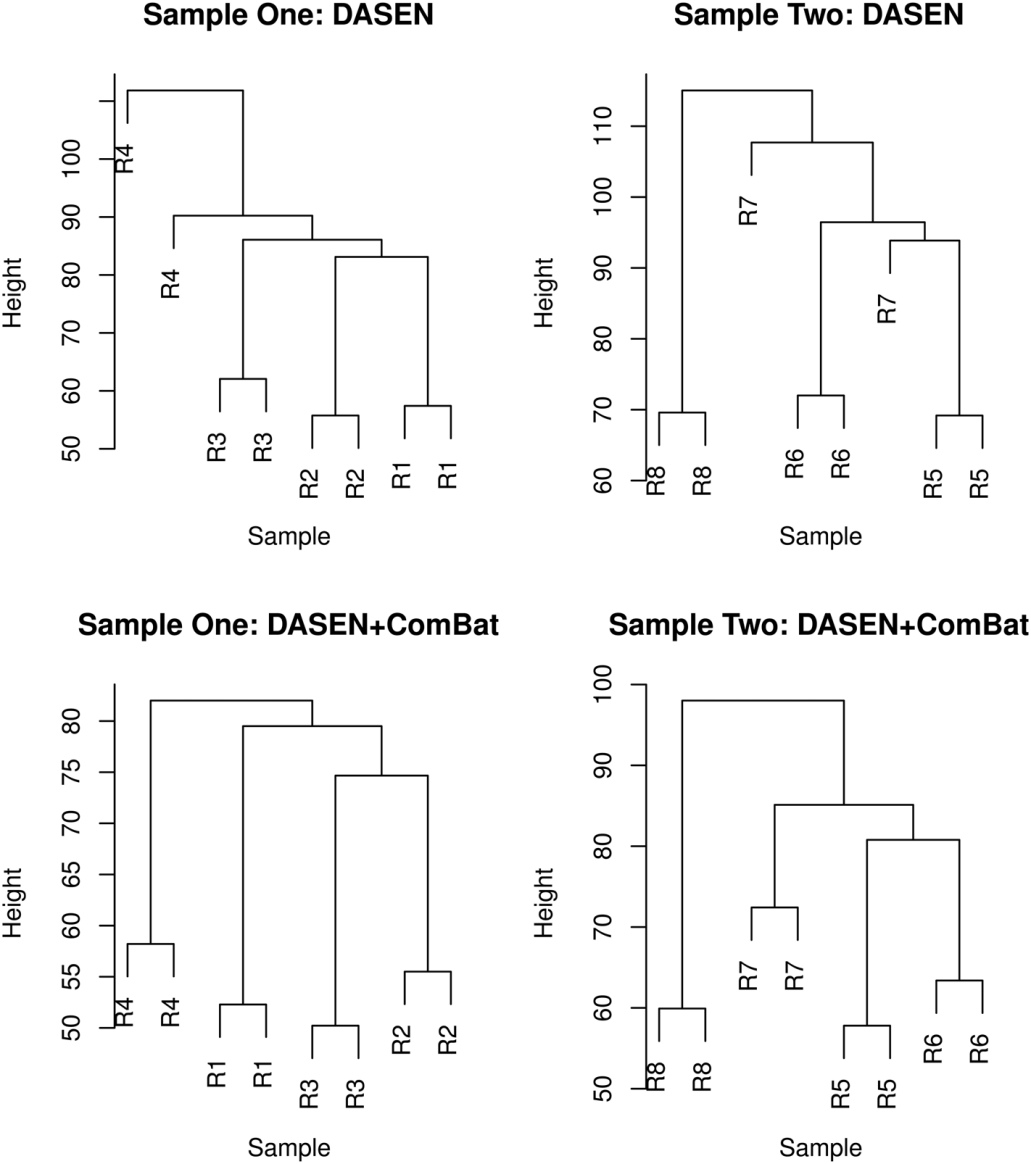
**Hierarchical clustering of technical replicates pre-ComBat (top row) and post-ComBat (bottom row)**. Duplicates are correctly paired post-ComBat in both samples.

The results in Figures 5, 6 showed that DASEN normalization followed by adjustment, using ComBat, for chip and row effects lead to greater reduction in variability among the technical replicates. Furthermore, the MDS plots (Figure 7) and the hierarchical clustering trees (Figure 8) both indicated correct and closer pairing of the technical replicates after ComBat.

**Figure 9:**
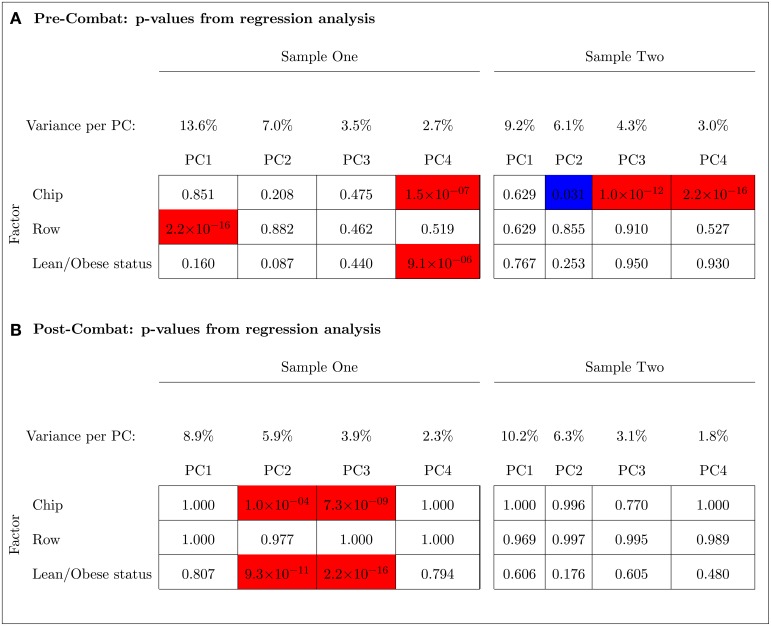
**Principal Component Analysis shows that ComBat effectively removed the row effect but not the chip effect in Sample One (unbalanced); however, it effectively removed the chip effect in Sample Two (balanced)**. Blue and red represent *p*-values < 0.05 and < 0.001, respectively. The percent variance explained by each principal component is shown. **(A)** Pre-ComBat: *p*-values from regression analysis. **(B)** Post-ComBat: *p*-values from regression analysis.

The PCA results in Figure 9 indicated that ComBat effectively removed row effects but not chip effects in Sample One. Before adjusting for chip and row effects using ComBat, the fourth and the first PCs were, respectively, significantly associated with chip and row (*p* < 0.001). The fourth PC was also significantly associated with the outcome variable (lean/obese status) (*p* < 0.001). After using ComBat, the second and third PCs were significantly associated with chip effects (*p* < 0.001) but no PCs were associated with row effects. The second and third PCs were also significantly associated with the outcome variable (*p* < 0.001). Thus, ComBat increased the number of PCs significantly associated with the chip batch and the outcome variable.

In contrast to Sample One, ComBat was able to effectively remove the chip effect in the balanced Sample Two (left panel of Figure 9). Before ComBat, the second, third, and fourth PCs were significantly associated with the chip batch at *p*-values < 0.05, < 0.001, < 0.001, respectively. After ComBat, no PC was found to be significantly associated with the chip batch. The row batch and the outcome variable were not significantly associated with any of the PCs before and after using ComBat.

### Differential methylation analysis

A total of 469,507 (Sample One) and 469,328 (Sample Two) CpG loci across the genome were tested in two sets of 92 individuals (excluding duplicates). For Sample One, a total of 25,650 and 94,191 probes emerged as differentially methylated when using the Pre-ComBat and Post-ComBat *M*-values, respectively, at an *q*-value threshold of 0.05 (Table 2). In addition, 369 of 25,650 and 3660 of 94,191 probes had *p*-values less than the Bonferroni threshold of 1.06 × 10^−7^. In marked contrast, Sample Two had zero differentially methylated probes between the obese and lean males when using either the Pre-ComBat or the Post-ComBat *M*-values (Table 2).

**Table 2:** **Differential methylation analysis results**.

	**Sample One**		**Sample Two**	
	**Pre-ComBat**	**Post-ComBat**	**Pre-ComBat**	**Post-ComBat**
Total CpGs sites tested	469,507	469,507	469,328	469,328
Number of sites identified at *q*-value threshold of 0.05	25,650	94,191	0	0
<Bonferroni threshold 1.06 × 10^−7^	369	3,660	0	0

## Discussion

Batch effects in high-throughput experiments that include but are not limited to chip position and run dates are a common and powerful source of variation in DNA methylation arrays. When these batch effects are confounded with the variables of interest, they can lead to inaccurate conclusions. In this study, we examined the impact of batch effects, in particular the chip and row in which the samples were assayed, on the number of differentially methylated probes when the study design is flawed. We had two study groups (Sample One and Sample Two) sampled from the same population and with similar demographic characteristics (Table 1). For Sample One, the lean were plated first followed by the obese, while for Sample Two the samples were balanced ensuring equal allocation of the lean/obese status, census region and age group (Figure 1). Given the small sample sizes, a balanced design is more appropriate than a randomization scheme employed by Harper et al. (2013). Simple randomization is known to lead to imbalanced group sizes or clustering in small studies. Lachin et al. (1988) showed that for small studies (i.e., *n* < 100 overall or within any principal group), imbalances that might affect power are more likely with complete or simple randomization. Balanced designs discovered by William S. Gosset aka “Student” on the other hand are known to be more powerful and hence are more efficient than randomized designs (Ziliak, 2014). A balanced design tries to control for factors that may be confounded with the outcome of interest hence leading to more valid inferences than those from a completely randomized design. Thus, a balanced design was chosen to reduce confounding of the chip and row batch effects with the outcome variable of interest (lean/obese status) which was evident in Sample One.

Although we did our best to adjust for chip- and row-specific batch effects from both datasets, ComBat was not able to remove chip-specific batch effects from the unbalanced Sample One. Moreover, the number of differentially methylated probes between the obese and lean (Table 2) increased from 5% (Pre-ComBat) to 20% (Post-ComBat) in Sample One compared to 0% in Sample Two (both Pre- and Post- ComBat). It seems unlikely that the slight demographic differences between Sample One and Sample Two (Table 1) would lead to such a dramatic difference in the number of differentially methylated probes. In addition, since normalization by itself cannot remove batch effects, we would expect to see similar results even if we tried a different normalization scheme. It is likely that the dramatic differences seen in Table 2 are caused by batch effects and that the results from Sample One are unreliable. While it is beyond the scope of this current work, it would be of interest to directly measure methylation using other technologies to confirm that these differences are the results of batch effects. Alternatively, similar to what Harper et al. (2013) have done, one could run the same samples twice, once with an unbalanced design and once with a balanced design.

Even with these batch effects we still may be able to make genetic inferences from these data. For instance, Spielman and Cheung (2007), in their response to Akey et al. (2007), noted that if we can find a genetic marker that determines the level of methylation, then we should see differential methylation between the case and control groups when stratified by genotype. That is, a batch effect should apply to everyone, even within each genotype sub-group, enabling us to distinguish between batch effects and genetic effects on methylation levels.

## Conclusion

Our results illustrate that experimental design is crucial in guarding against batch effects in 450K methylation arrays. When the variables of interest are confounded with batch effects, it is very difficult to separate them using sophisticated and careful statistical analysis. Although our results agree with those of Harper et al. (2013) who found a reduced number of significant probes when they randomized their samples on the chips, our design of balancing samples across the chips and rows is more optimal than randomization given the small sample size. Our design removes any clustering that could again confound results, which is more likely with the randomization scheme employed by Harper et al. (2013). In general, adjusting for batch effects using ComBat leads to improved agreement between technical replicates, and an increased number of differentially methylated sites (DMPs) (Harper et al., 2013). However, these DMPs should be taken with caution if the samples are not balanced across the chips by the variable of interest and any other variables that could easily confound the final results.

Our results imply that great care should be taken when designing a study so as to guard against possible batch effects. While this need has been prominently discussed previously (e.g., Leek et al., 2010), we must be ever vigilant to ensure good communication between the statistical members of the research team and the laboratory running the methylation arrays. Batch effects can not only lead to false positive signals, but, if not corrected for adequately, could obscure important true positive signals (Baggerly et al., 2004; Akey et al., 2007; Leek and Storey, 2007; Harper et al., 2013). As Light et al. (1990) wrote: *“You can't fix by analysis what you bungled by design.”*

## Author contributions

Olive D. Buhule analyzed the data and wrote the initial draft of the manuscript. Nicola L. Hawley and Stephen T. McGarvey did the fieldwork in Samoa with the assistance of Satupaitea Viali, collecting the phenotypic data and the blood samples. Daniel E. Weeks supervised the statistical analyses. Ryan L. Minster assisted Olive D. Buhule with the details of using R and LaTeX. Mario Medvedovic did initial quality checks of the data. Ranjan Deka supervised Guangyun Sun as he processed the blood samples and prepared them for analyses by the core laboratory. Stephen T. McGarvey, in close collaboration with Daniel E. Weeks and Ranjan Deka, originated and directed the study. All authors contributed to the writing of the manuscript.

## Funding

This work was supported by the NIH grant R01 HL093093 (PI: Stephen T. McGarvey).

### Conflict of interest statement

The authors declare that the research was conducted in the absence of any commercial or financial relationships that could be construed as a potential conflict of interest.
